# Portal vein thrombosis after cetuximab and 5-fluorouracil therapy in a patient with advanced colon cancer and decompensated cirrhosis: a case report and review of the literature

**DOI:** 10.1186/s12957-023-03175-7

**Published:** 2023-09-23

**Authors:** Fangyu Liu, Yongmei Xu, Guowang Yang, Yuhong Guo, Jiayun Nian

**Affiliations:** Beijing Hospital of Traditional Chinese Medicine, Capital Medical University, Beijing, China

**Keywords:** Advanced colon cancer, Decompensated cirrhosis, Cetuximab, 5-fluorouracil, Portal vein thrombosis

## Abstract

**Background:**

Treatment options for advanced colon cancer are mainly combinations of chemotherapy and targeted drugs. However, poor physical health and medication intolerance limit the choice of anticancer drugs. Colon cancer with cirrhosis is a particular patient group that poses a challenge to clinical treatment.

**Case presentation:**

This article presents a case of a patient in the decompensated stage of cirrhosis who was diagnosed with advanced colon cancer. The initial presentation was a nodule on his navel named the Sister Mary Joseph’s nodule, which was later confirmed by biopsy and PET-CT as one of the metastases of colon cancer. The patient was treated with cetuximab and 5-fluorouracil at a below-guideline dose; however, portal vein thrombosis developed and led to death. This entire process, from diagnosis to death, occurred within a span of three months.

**Conclusion:**

Cancers with cirrhosis are a special group that deserves more attention. There is no unified treatment guideline for these patients, especially those with extrahepatic primary tumors. We should be more cautious when choosing treatment for such patients in the future. Both chemotherapy and targeted treatment may potentially induce portal vein thrombosis, which appears to have a higher incidence and worse prognosis than cancers without cirrhosis.

## Background

Colon cancer is the fifth most common cancer in the world [1]. Due to the lack of specific clinical manifestations and screening tools, many cases of colon cancer are only diagnosed at an advanced stage. The 5-year survival rate of patients with advanced colon cancer is 14% [2]. Treatment options for advanced colon cancer should be stratified based on various factors, including the location of the primary lesion, microsatellite and mismatch repair status, and the presence of RAS and BRAF gene mutations [3]. However, the physical state of the patients may limit the available treatment options to some extent.

Chronic hepatitis is a persistent global public health burden that can cause cirrhosis and cancer, including primary liver cancer and extrahepatic solid tumors [4, 5]. According to GLOBOCAN 2012 [6], 15.4% of new cancer cases are attributable to carcinogenic infections, among which hepatitis B and C viral infections account for 19.2% and 7.8% of cases, respectively. However, there is a lack of consensus regarding the treatment of patients with tumors associated with viral hepatitis and cirrhosis, especially those with extrahepatic solid tumors.

This article reports a rare and challenging case of advanced RAS and BRAF wild-type colon cancer combined with cirrhotic decompensation. The patient developed portal vein thrombosis following dose reduction therapy with cetuximab and 5-fluorouracil and eventually died within 3 months of cancer diagnosis. This case highlights an important clinical dilemma regarding the management of patients with cancer and cirrhosis, as standard antitumor therapies may lead to unforeseen complications.

## Case presentation

A 61-year-old man with a 40-year history of chronic hepatitis B developed cirrhosis, splenomegaly, and hypersplenism over a period of 20 years. The patient underwent surgery for ligation of the esophageal and gastric varices 10 years ago and laparoscopic gallbladder removal with stones 6 years ago. In 2021, a 5-mm nodule was discovered at the scar site on the navel, which gradually increased to 4 cm. Subsequently, the nodule was resected under local anesthesia on May 30, 2022. Pathological examination revealed dermal and subcutaneous adenocarcinoma with intravascular cancer embolus, raising the possibility of a primary gastrointestinal tumor or a tumor originating from the umbilical ureteral site. Immunohistochemical analysis revealed the following results: CDX2 ( +), CK19 ( +), CK7 ( −), SATB2 ( +), CD31 (vessel, +), PMS2 ( +), MLH1( +), MSH6 ( +), MSH2 ( +), HER2 (20%, 2 +), PD-L1 (tumor cells − , immune cells 15% +) and no gene mutations detected in KRAS, NRAS, PIK3CA, and BRAF genes. To further evaluate the diagnosis, PET-CT scan was performed on June 13, 2022. The scan showed localized thickening of the intestinal wall in the sigmoid colon, along with abnormal glucose metabolism. The affected segment of the intestinal wall measured approximately 3.7 cm in length, with a maximum standardized uptake value (SUVmax) of 12.9, indicating a potential malignant lesion with multiple surrounding metastatic lymph nodes. Multiple nodular foci of increased glucose metabolism in both lungs were considered malignant lesions, and a localized increase in glucose metabolism was observed after resection of the umbilical masses. Due to the patient’s low platelet count of 30 × 10^9^/L, the colonoscopy had to be interrupted. Through multi-disciplinary team (MDT), the patient was diagnosed with stage IV sigmoid colon cancer with lung, pelvic lymph node, and abdominal wall metastases.

The patient underwent a pre-medical evaluation and preparation on June 21, 2022. The evaluation results showed a Child–Pugh score of 9 (Grade B), splenomegaly, hypersplenism, and esophagogastric varicose veins, suggesting poor liver reserve and decompensation of liver cirrhosis. Moreover, the patient had a low count of neutrophils and thrombocytes (WBC 2.61 × 10^9^/L; PLT 44 × 10^9^/L). The patient received recombinant human granulocyte-stimulating factor injection (rhG-CSF) (0.1 mg QD) and recombinant human thrombopoietin injection (rhTPO) (15,000 U QD) once a day from June 27 to July 6, 2022. On recheck on July 7, the white blood cell count increased to 4.55 × 10^9^/L and the platelet count rose to 64 × 10^9^/L. With informed consent, the first chemotherapy combined with targeted therapy was administered at a below-guideline dose: cetuximab 600 mg (320 mg/m^2^) d1, 300 mg (160 mg/m^2^) d8 + Calcium Folinic Acid 760 mg (400 mg/m^2^) d1 + 5-fluorouracil 500 mg (366 mg/m^2^) via intravenous infusion for 2 h followed by a 3000-mg (1600 mg/m^2^) infusion pump for 48 h every 14 days.

**Fig. 1:**
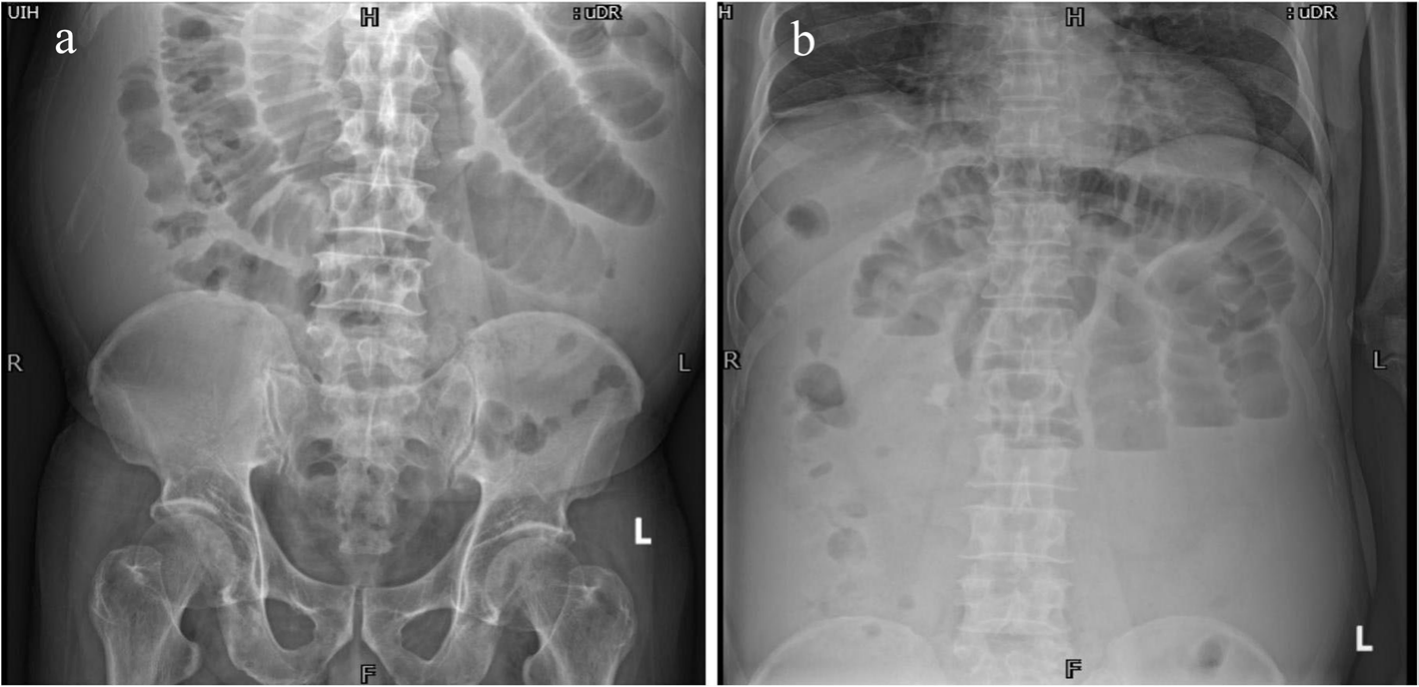
Plain radiography of the abdomen. **a**, **b** The small intestine has multiple gas–liquid levels with intestinal dilatation

**Fig. 2:**
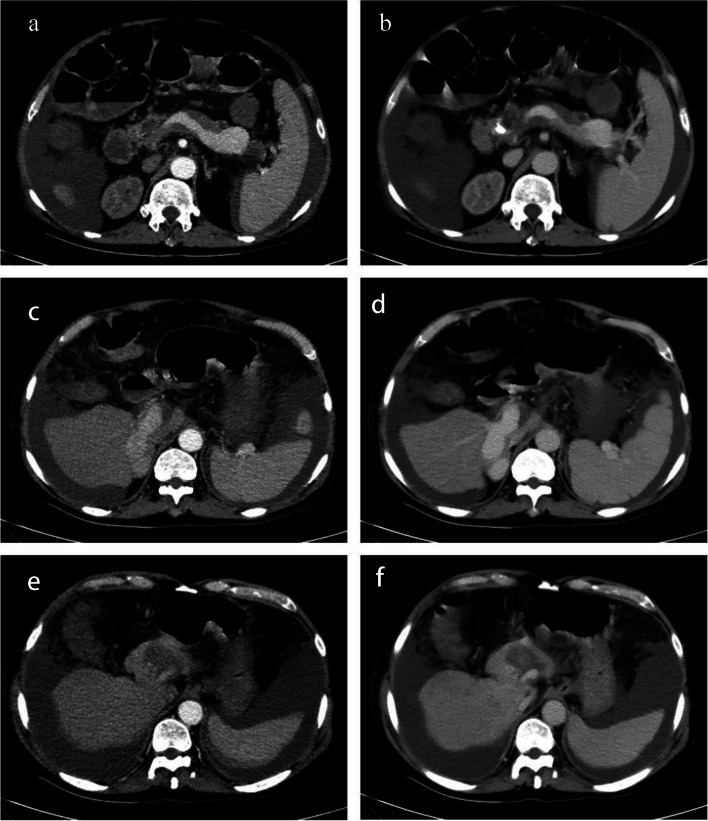
Enhanced abdomen CT. **a**, **b** Filling defects of splenic vein. **c**, **d** The right branch of the portal vein appears thickened. **e**, **f** Filling defects of left branch of the portal vein

Treatment was initiated on July 8; however, on July 10, the patient developed abdominal pain during 5-fluorouracil pumping, followed by abdominal distention. Analgesics were administered for pain relief, and 5-fluorouracil pumping and subsequent treatments were discontinued. On July 11, the patient experienced progressively worsening abdominal distension and pain, along with over twenty episodes of bowel movements. Montmorillonite powder and loperamide were administered to treat the diarrhea. On the night of July 11, the patient developed a fever with a maximum temperature of 38 °C. Blood tests indicated a significant increase in leukocytes levels (14.52 × 10^9^/L), absolute neutrophil value (13.62 × 10^9^/L), and platelet count (200 × 10^9^/L). Ceftazidime was administered intravenously at a dose of 1 g every 8 h for empirical anti-infection. The patient had no diarrhea since 17:00 on July 12; however, abdominal distension and pain had significantly worsened. Abdominal radiography revealed multiple air-fluid planes in the small intestine and slight intestinal dilation, indicating small intestine obstruction (Fig. 1). Abdominal ultrasonography revealed a large volume of peritoneal fluid with a maximum depth of 12.2 cm. The patient underwent fasting and water deprivation, laxative enema, human albumin infusion, and liquid management; however, the symptoms of abdominal distension did not improve significantly. Enhanced abdominal CT on July 15 showed filling defects in the portal and splenic veins without significant enhancement (Fig. 2). Blood coagulation showed a D-dimer level of 7.28 mg/L. After a multi-disciplinary team evaluation, the patient received anti-coagulation therapy with an injection of low-molecular-weight heparin 10,000 IU per day (1300 U/kg/day, 6000 IU in the morning, and 4000 IU in the evening), considering the high risk of thrombolysis or vascular intervention to remove the embolus. Transesophageal gastrotubing was simultaneously performed to reduce nausea and vomiting. Endoscopic findings included esophageal varices, along with thickening and hardening of the intestinal wall starting from a distance of 20 cm from the anal verge. The intestinal lumen exhibited narrowing, making it impossible for the endoscope to pass through. Biopsy was not performed to avoid bleeding. Due to further deterioration, the patient suffered from an abdominal infection, bacterial and fungal pneumonia, and respiratory failure, which led to death on August 9.

## Discussion and conclusions

This paper reports a case of advanced sigmoid colon cancer combined with cirrhotic decompensation that developed into portal and splenic vein thromboses after cetuximab combined with 5-fluorouracil reduction therapy. There was less than 3 months of survival from diagnosis to death. This is a rare and challenging complication.

This patient was first diagnosed with an unusual and rare metastatic nodule on the navel, named the Sister Mary Joseph nodule (SMJN) [7]. SMJN are characterized as tumor lesions that manifest on the navel and can present as primary tumors or metastatic sites of visceral malignancies tumors, including pancreatic, gastrointestinal, and gynecological tumors, with a total incidence of 1–3% [8–11]. Metastatic lesions may spread to the navel through the lymph ducts, venous network, contiguous extension from adjacent areas, embryological remnants, or as a result of iatrogenic surgery [12]. The presence of metastasis in the belly button is often associated with a poor prognosis, with a median survival of 28 weeks [8].

The combination of malignant tumors and cirrhosis, especially in the decompensated stage, poses a major challenge to clinicians. This is common in patients with primary hepatocellular carcinoma, approximately 90% of whom are in the setting of cirrhosis [13]. Portal vein thrombosis is a rare event in the general population but occurs relatively frequently in patients with cirrhosis. The incidence of portal vein thrombosis was 13.9% in patients with cirrhosis [14], however, increases to 35% in patients with hepatocellular carcinoma [15]. The main therapeutic objectives of liver cirrhosis with portal vein thrombosis are to control or prevent complications by restoring blood flow and preventing further thrombosis. This can be achieved, using anti-coagulation drugs, interventional techniques, and surgical interventions [16, 17]. Portal vein thrombosis may lead to serious complications, including mesenteric ischemia or infarction, particularly in patients with decompensated cirrhosis, resulting in high mortality rates. However, managing portal vein thrombosis becomes more challenging in patients with concurrent tumors. Clinicians not only need to control the thrombus but also conduct antitumor therapy. It is important to consider that both the tumor itself and the antitumor therapy may induce or aggravate thrombus formation.

There is a lack of recommended treatment options according to the guidelines for patients with colon cancer combined with decompensated cirrhosis. Such patients are often excluded from clinical trials, and currently, most similar patients are recorded in the form of case reports [18–20]. Despite careful selection and dose reduction of antineoplastic drugs, portal vein thrombosis occurred, eventually leading to the patient’s death. Portal vein thrombosis occurs in the main trunk or branches of the portal vein and can cause increased portal pressure and intestinal stasis. Clinical manifestations of portal vein thrombosis include abdominal pain, bloating, vomiting, diarrhea, peritonitis, paralytic intestinal obstruction, and massive ascites. In the present case, the patient presented with related symptoms. Not all vein thromboses are life-threatening, and their prognosis partly depends on the degree of vessel occlusion. The higher the degree of occlusion, the worse the pathogenic condition, and it is clear that this patient had a high occlusion.

The etiology of portal vein thrombosis is complex and includes decreased portal flow velocity, increased severity of cirrhosis, portal venous endothelial injury and inflammation [21–23]. The formation of portal vein thrombosis in this patient occurred post-treatment; therefore, there is sufficient reason to suspect a close relationship between thrombosis and the therapy. However, we do not yet have enough evidence to point out which is the more important cause of thrombosis, 5-fluorouracil or cetuximab. Both drugs are associated with thrombosis, including deep vein and artery thrombosis [24, 25]. Cetuximab, as an anti-EGFR /HER monoclonal antibody, has antiangiogenic activity [26], which is thought to be a possible cause of thrombosis. Fluorouracil, on the other hand, may induce thrombosis through mechanisms involving vascular endothelial injury, resulting in vasoconstriction and spasms [27]. However, in this case of advanced colon cancer and cirrhosis, the cause of thrombosis was more likely the result of multiple factors, which may be related not only to anti-tumor treatments but also to the underlying physical condition.

Clinicians should be cautious when administering chemotherapy or targeted therapy to patients with tumors and cirrhosis. The potential risk of thrombosis and bleeding should be considered in advance and fully evaluated (e.g., Caprini, Padua, or IMPROVE score), especially if the patient is at an advanced stage of the tumor or has decompensated cirrhosis. For patients with a high risk of thrombosis and a low risk of bleeding, it is necessary to initiate preventive measures as early as possible, including low molecular weight heparin (LMWH) or direct oral anticoagulants (DOACs) [28]. However, this patient has a high risk of thrombosis(Caprini Score = 4), but also a high risk of bleeding(IMPROVE score = 8.5), the risks and benefits of anticoagulant need to be fully weighed, which is obviously a very difficult decision to make, but mechanical prophylaxis can be an alternative. Antitumor therapy may be considered for patients with tumors and cirrhosis who are at low risk of thrombosis, or who are at high risk of thrombosis but have taken anticoagulants prophylactically. However, it is recommended to first chose palliative measures for patients who are at high risk of thrombosis and bleeding and do not use anticoagulant prophylactically. In addition, it is necessary to be familiar with the typical symptoms of intestinal ischemia and necrosis after superior mesenteric vein thrombosis to identify such patients as early as possible and conduct interventions immediately after diagnosis unless contraindicated.

## Data Availability

The original contributions presented in the study are included in the article. Further inquiries can be directed to the corresponding authors.
